# Coverage of complex tissue defects following open cervicothoracic spine surgery with a lower trapezius island myocutaneous flap—an interdisciplinary approach

**DOI:** 10.1007/s10143-021-01621-2

**Published:** 2021-08-18

**Authors:** Armin Osmanagic, Alessa Schütz, Ivo Bayard, Andreas Raabe, Radu Olariu, Ralph T. Schär

**Affiliations:** 1 Department of Neurosurgery, Inselspital, Bern University Hospital, University of Bern, Freiburgstrasse 16, 3010 Bern, Switzerland; 2 Department of Plastic and Hand Surgery, Inselspital, Bern University Hospital, University of Bern, Bern, Switzerland

**Keywords:** Spine surgery, Surgical site infection, Trapezius island myocutaneous flap, Wound healing disturbance

## Abstract

The study design is a clinical case series. The objective of this study was to present the concept and efficacy of the lower trapezius island myocutaneous flap (LTIMF) for management of complex wound healing disorders following open cervicothoracic spine surgery. Wound healing disturbances with myocutaneous defects after open spine surgery at the cervical and upper thoracic spine are well-described complications. In severe cases, plastic reconstructive coverage is often required as a last resort. A review of all adult patients with deep wound dehiscence and tissue defects following open cervicothoracic spine surgery, who were managed with plastic surgery reconstruction using a LTIMF at our institution, was conducted. Synopses of these cases are presented. Seven patients with a mean age of 73 years ± 13 (range 50 to 89 years) were included in this case series. Six out of seven patients had instrumented posterior fusion added to their decompression. All patients were managed with a LTIMF for wound coverage. No spinal implants were removed prior to LTIMF surgery. The mean follow-up was 5.2 months (± 5.4 months). No major flap failure occurred, and all patients presented with satisfactory cosmetic results. The only minor complication was development of a sterile subcutaneous seroma in two patients, which were successfully managed by puncture and aspiration. The LTIMF is an effective and reliable salvage treatment option for spine surgery patients offering stable coverage of deep tissue defects resulting from complex wound healing disorders at the cervical and upper thoracic spine.

## Introduction

Deep wound dehiscence and surgical site infections (SSIs) are feared complications following open posterior spine surgery, especially at the cervical and upper thoracic spine. Management of deep wound dehiscence poses a difficult challenge for spine surgeons, especially in cases with exposed hardware. Treatment options include conservative management, debridement, systemic and local antibiotic therapy, and vacuum-assisted closure (VAC) dressings [1]. For patients with complicated wound defects, these strategies are usually prone to fail. Ultimately, for most of these patients, reconstructive surgery using an interdisciplinary approach is required for successful wound coverage. Well-vascularized musculocutaneous flaps have shown to provide reliable reconstruction of complicated wounds, due to active inhibition of bacterial growth in contaminated wounds [2]. Depending on the anatomical location, the most common flaps used for coverage of complex spinal wounds are paraspinal muscle, latissimus, and trapezius [3]. At our institution, a lower trapezius island myocutaneous flap (LTIMF) has been established as a salvage therapy to cover extensive tissue defects over the cervicothoracic spine. The LTIMF is a well-described pedicled flap offering a high flexibility of inset, and used for a variety of reconstructive procedures from oromaxillofacial to deep tissue defects over the spine. However, reports of the LTIMF for reconstruction of complicated wounds after spine surgery are limited to a few small case series (4 to 6 patients) and case reports, and find little mention in the spine literature [4–11].

In this study, we present a consecutive series of seven adult patients with complex wound healing disorders following open cervicothoracic spine surgery who were managed with a LTIMF.

The aim of this study was to demonstrate the feasibility and efficacy of the LTIMF in an interdisciplinary approach for management of complex soft tissue defects after spinal surgery at the cervicothoracic spine.

## Methods

### Study design and study population

This was a retrospective clinical case series. Written general consent was obtained from all patients included in this study. All patients with complex wound healing disturbances following surgery at the cervical and/or thoracic spine and treated with a LTIMF between November 2011 and October 2020 at our hospital were included in this case series. Patients’ medical records, surgical notes, and photo documentations, which were taken during their hospital stay, and all clinical follow-up notes, were reviewed.

### Surgical technique

The triangular-shaped trapezius muscle is the most superficial muscle of the back, and consists of a descending, transverse, and ascending part. The dominant blood supply of the LTIMF is the dorsal scapular artery (DSA), with the descending branch of the superficial cervical artery also contributing as a secondary pedicle. Although there is quite some anatomical variability in the origin of the DSA and the superficial cervical artery [12], this does not interfere with the relevant vascular anatomy for flap raising. The DSA always runs below the levator scapulae and pierces superficially between the rhomboideus major and minor to the trapezius muscle. The skin island is perfused through perforators piercing the muscle.

**Fig. 1:**
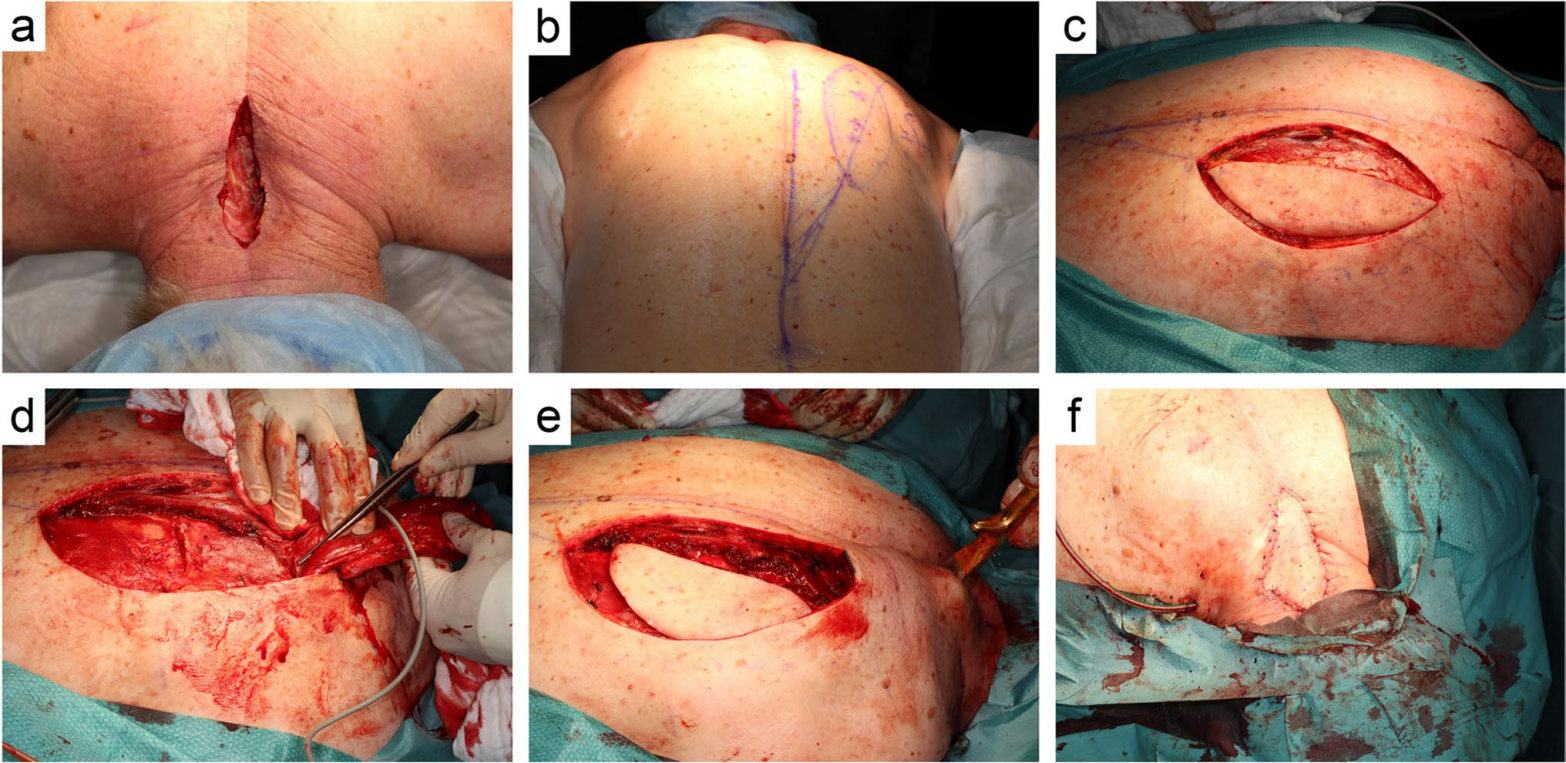
Surgical steps of the LTIMF procedure (case 6): **a** Wound after removal of VAC dressing. **b** Preoperative planning and markings of the flap. **c** Dissection of the myocutaneous flap. **d** Identification of the supplying dorsal scapular artery. **e** Rotation and tunneling of the flap to the cervicothoracic defect. **f** Flap is placed and sutured at the cervicothoracic junction. A subcutaneous drain is placed

Flap markings are made in the standing patient. The distal muscle origin is marked at the level of T12. Perforators to the skin can be marked with a hand-held Doppler in order to ensure that they are included in the skin paddle (Fig. 1b)*.* The skin paddle is marked slightly lateral to the midline and can be one-third distal to the edge of the muscle to extend the reach of the flap. To harvest the flap, the patient is usually placed in prone or alternatively lateral position. The skin paddle is then incised on the lateral side and the muscle is identified from lateral to medial (Fig. 1c). It is then dissected from distal to proximal preserving the DSA that can be identified on the deep surface of the flap (Fig. 1d). For defects of the cervical and cervicothoracic spine, the whole width of the muscle can be harvested, thereby including the descending branch of the superficial cervical artery for a more robust vascularization. The dissection should not exceed the scapular spine, as this may lead to functional impairment. A tunnel is then made between the harvest site and the defect (Fig. 1e) and the flap is passed through the tunnel. Care has to be taken to ensure adequate dimensions of the tunnel, as compression may lead to venous congestion of the flap. A low threshold for incising the tunnel all the way to the defect should be maintained if there is any suspicion of venous congestion. The flap is then sutured in the defect with the muscle obliterating any dead space. The skin paddle can be tailored to the needs of the defect, in order to ensure tension-free closure (Fig. 1f). A drain is placed at the donor and recipient site. In virtually all cases, the donor site can be closed primarily due to the horizontal laxity of the thoracic paravertebral skin.

### Case presentations

In the following, the seven cases are briefly summarized. More detailed information regarding patient history and possible risk factors is listed in Tables 1 and 2.

**Table 1:** Overview of patient demographics and potential risk factors for wound healing disturbances

Case	Age (years)	Sex	Diabetes mellitus	Smoking	RT prior of after index surgery	Cumulative RT dose (Gy)	History of surgery at index level
1	50	M	No	No	4 weeks prior	12	No
2	68	M	Yes	No	None	-	Yes
3	89	F	No	No	12 weeks prior	30	Yes
4	67	M	No	No	2.5 weeks after	30	No
5	74	M	No	Yes	None	-	Yes
6	86	M	No	No	None	-	No
7	74	F	No	No	None	-	Yes
Mean or rate	73 ± 13	71.4%	14.3%	14.3%	42.9%	24 ± 10.4	57.1%

*M* male, *F* female, *SD* standard deviation, *RT* radiation therapy, ± standard deviation

**Table 2:** Overview of surgical information

Case	Indication	Level of surgery/instrumentation	Index surgery duration (min)	Time to SSI (days)	SSI level	Bacteriological profile	Time between SSI and LTIMF (days)	Last FU (months)
1	Tumor	C4-T6	272	31	T5-7	Coagulase-negative *Staphylococcus*	2	0.7
2	Degenerative	C3-7	305	29	C3-7	Coagulase-negative *Staphylococcus*	2	15.3
3	Tumor	C3-T2	187	23	C3-T2	Cultures w/o bacterial growth	19	1.1
4	Tumor	C4-T4	232	88	C4-T4	Cultures w/o bacterial growth	30	8.1
5	Degenerative	C2-5	185	13	C4	*Propionibacterium acnes*	12	7.2
6	Hematoma	C2-T6 w/o instrumentation	235	85	C5-7	Cultures w/o bacterial growth	6	2.2
7	Degenerative	C3-T2	216	22	C3-T2	Coagulase-negative *Staphylococcus*	8	1.6
Mean or rate	-	-	233 ± 44	42 ± 31	-	57%	11 ± 10	5.2 ± 5.4

*LTIMF* lower trapezius island myocutaneous flap, *SSI* surgical site infection, *w/o* without, ± standard deviation

#### Case 1

A 50-year-old man suffered pathological vertebral body fractures at C6 and T3 due to a metastasized pulmonary adenocarcinoma with consecutive spinal canal stenosis and spinal cord compression. He underwent decompression and dorsal stabilization from C4 to T6. Four weeks postoperatively, the patient underwent adjuvant, palliative radiation therapy with a total radiation dose of 12 Gray (Gy). Five days into radiation therapy, a wound dehiscence of 9 × 5 cm occurred with evident deep SSI. Cultures from the wound revealed coagulase-negative staphylococci. The skin defect was covered with a right-sided LTIMF under targeted antibiotic therapy. No further postoperative complications occurred. However, the patient passed away 4 months postoperatively due to his underlying oncological diagnosis.

#### Case 2

**Fig. 2:**
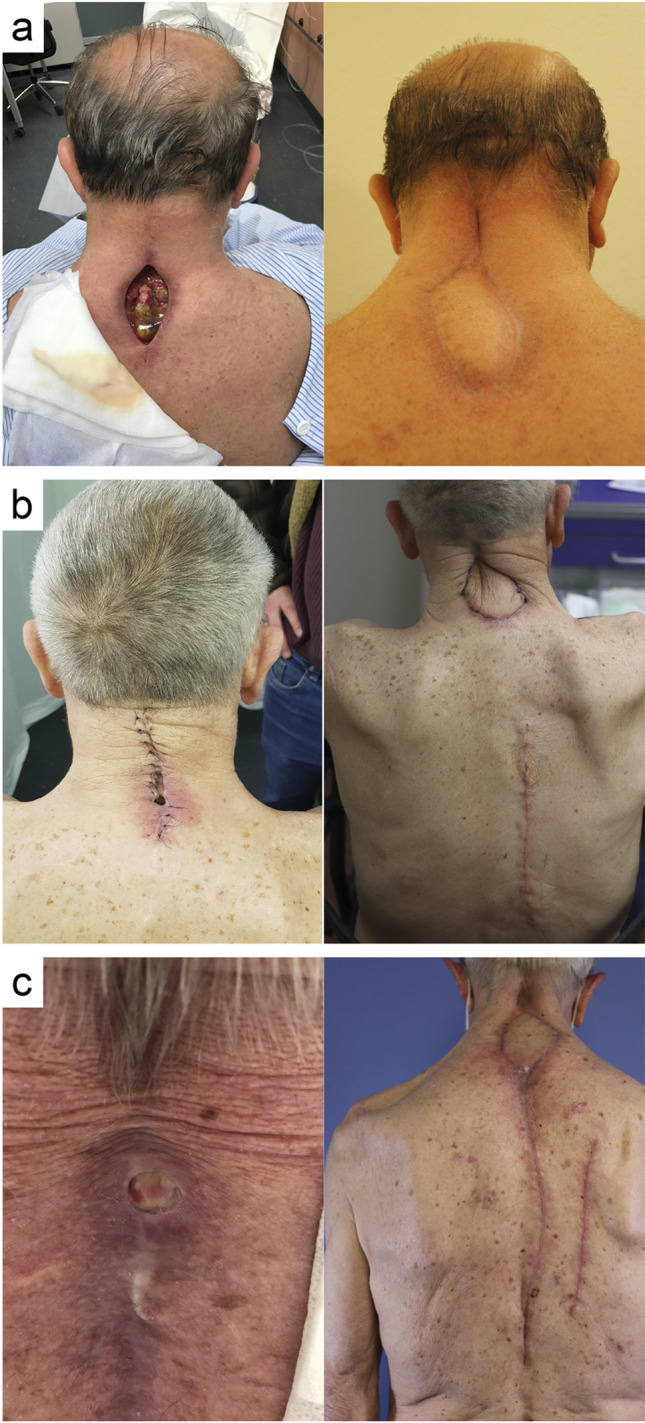
Clinical pictures showing preoperative wound dehiscence (left) and postoperative result (right) at last follow-up after LTIMF coverage of patients no. 2 at 4.3 months (**a**), no. 5 at 1 month (**b**), and no. 6 at 1.3 months (**c**)

A 68-year-old male patient underwent dorsoventral decompression and instrumented fusion from C3 to C7 due to degenerative cervical myelopathy. Four weeks postoperatively, he presented with a nuchal deep wound dehiscence with exposed spinous processes and implants (Fig. 2a). The wound was surgically debrided and a VAC dressing was applied. Coagulase-negative staphylococci were cultured from the wound. In a second step, and under targeted antibiotic therapy, the 12 × 4 cm sized tissue defect was covered with a LTIMF from the left side. At 3-month follow-up, a subcutaneous seroma at the caudal end of the flap was noted. It was punctured and 25 ml of sterile fluid was aspirated. Further follow-ups after 4 and 5 months showed no further evidence of seroma formation with satisfactory wound healing.

#### Case 3

An 89-year-old woman with an osteolytic bone lesion of C5 due to multiple myeloma with consecutive compression of the spinal cord underwent ventral corpectomy. Adjuvant radiation therapy (total dose of 30 Gy) was performed. Three months later, the patient received stabilization of C3 to T2 due to a pathological fracture of C4. Three weeks postoperatively, she presented with a deep posterior wound dehiscence of 7 × 12 cm with exposure of bony elements of the spine. Under empiric antibiotic therapy, a VAC dressing was applied. The defect was covered 18 days later with a LTIMF. At 2-month follow-up, she presented with a satisfactory surgical result without any complications.

#### Case 4

A 67-year-old man with metastasized adenocarcinoma of the rectosigmoid junction developed a pathological fracture at the cervicothoracic junction with compression of the C7 and C8 nerve roots. The patient was treated with decompression and dorsal stabilization from C4 to T4, followed by adjuvant radiation therapy (total dose of 30 Gy). Two months after radiation therapy, the patient developed a wound healing disturbance. Under empiric antibiotic therapy and prior VAC dressing over 4 days, the tissue defect of 9 × 4 cm was covered with a LTIMF. The flap healed well and no further complications occurred up to his last follow-up visit 1 year postoperatively.

#### Case 5

A 74-year-old man suffered a fall and subsequent traumatic C1 and C2 fracture, requiring a dorsal C1-2 fixation. Nine months later, he required laminectomy of C3 and dorsal stabilization to C4 for adjacent segment disease. Two weeks postoperatively, the patient presented with a deep wound dehiscence. Cultures taken from the wound revealed *Propionibacterium acnes*. Under targeted antibiotic therapy and prior VAC dressing over 12 days, the tissue defect was covered with a 5 × 15 cm LTIMF. At 1-month follow-up, he presented with a subcutaneous seroma of 15 ml, which was aspirated under local anesthesia. Otherwise, there was satisfactory wound healing without further complications (Fig. 2b).

#### Case 6

An 86-year-old patient developed a non-traumatic extensive spinal epidural hematoma, which was initially addressed with left-sided hemilaminectomies from C2 to T6 and hematoma evacuation. Due to early rebleeding in the lower thoracic epidural space, hemilaminectomies were extended down to T12 for hematoma evacuation. Three months later, the patient developed a wound healing disorder with a 1-cm wound dehiscence at the cervical spine. Under empiric antibiotic therapy and VAC dressing over 3 days, the complex tissue defect was managed with a LTIMF of 5 × 15 cm. At 2-month follow-up, the patient presented with satisfactory wound healing and no complications (Fig. 2c).

#### Case 7

A 73-year-old woman underwent C4-7 anterior cervical discectomy and fusion for degenerative cervical myelopathy. Four months later, failure of the anterior construct and progressive cervical kyphosis were noted. She was revised by means of C5-6 laminectomies and a C3-T2 posterior instrumented fusion. Three weeks later, she presented with a deep SSI with a 9-cm-long wound dehiscence over the cervicothoracic junction with exposure of the spinous processes. Cultures taken from the wound showed evidence of coagulase-negative staphylococci. Targeted antibiotic therapy was initiated, the wound was debrided, and a VAC dressing was applied. Two weeks later, the tissue defect was covered with a LTIMF. At 2-month follow-up, wound healing was satisfactory with no complications*.*

## Results

Within the 10-year observation period, 1338 spine surgeries at the cervicothoracic junction were performed at our institution. Postoperative wound healing disorders or SSIs were recorded for 66 patients (4.9%). Eleven of these patients were treated with myocutaneous muscle flaps, including the seven LTIMFs presented herein. The seven patients in this series aged 50 to 89 years and had a mean age of 73 ± 13 years. Indications for initial spine surgery comprised degenerative disease (*n* = 3), metastatic disease (*n* = 3), and a non-traumatic epidural hematoma (*n* = 1). Six patients received instrumented fusion, and the mean duration of the initial procedure was 233 min (± 43 min) with a range from 185 to 305 min. Five patients had surgery at the cervicothoracic junction, with two cases involving only the cervical spine. Preexisting potential risk factors present in our cohort were diabetes (*n* = 1), smoking (*n* = 1), prior spinal surgery at the same level (*n* = 4), and prior or postoperative radiation therapy (*n* = 3) at the surgical level. Five out of seven patients had culture-positive SSIs (three cases with coagulase-negative staphylococci, one case with *Propionibacterium acnes*). The mean latency to the occurrence of the wound dehiscence following the index surgery was 37 (± 31, range 8–88) days, and surgical coverage was performed at a mean of 15 (± 14, range 2–41) days after wound dehiscence and SSI occurrence. Application of a VAC dressing prior to the LTIMF was applied in six out of seven cases. In none of the seven cases were spinal implants removed or exchanged prior to LTIMF coverage. The mean clinical follow-up was 5.2 ± 5.4 months after surgery (range 0.7 to 15.3 months). All patients presented with satisfactory wound healing results at their last follow-up visit, and none of the patients experienced flap failure or other major complications. Two patients (cases 2 and 5) developed a small seroma under the flap, which was aspirated under local anesthesia.

## Discussion

In our series of seven patients with complex wound healing disturbances after open cervicothoracic spine surgery with instrumentation in most cases, coverage using a LTIMF leads to successful wound healing in all cases without any flap failures observed during follow-up. Of note, no implants had to be removed prior to flap coverage. Our results concur with previous reports in the literature with excellent functional and cosmetic outcomes [5–9].

In our practice, we generally adhere to a staged management concept. In a first stage, surgical wound debridement with microbiological sampling is performed. Complex and deep wound defects are usually temporarily covered with a VAC dressing. An interdisciplinary case discussion involving spine surgeons, plastic surgeons, and infectious diseases specialists takes place to reach a consensus regarding the optimal patient management. Also, the necessity of hardware removal or exchange thereof is discoursed. Under targeted antibiotic therapy for a predefined duration, the wound is finally covered with a LTIMF in complex cases in which wound debridement and primary closure are not feasible.

Recognizing and mitigating potential risk factors in order to prevent the occurrence of wound dehiscence and SSIs is of utmost clinical importance. In spine surgery, SSIs have been shown to be associated with higher morbidity and mortality, lead to reoperation, and prolonged hospitalization [13]. Chieng et al. performed a systematic review and pooled analysis of 262 spine surgery patients showing surgery involving instrumentation, history of radiation therapy, smoking, and diabetes mellitus to be risk factors for the development of complex wound healing disorders requiring muscle flap coverage [14]. In our series, only one patient had a history of smoking, and only one other patient had diabetes. Conversely, the majority of our patients had undergone instrumented fusion, and had been exposed to radiation therapy before or after their spine surgery. When operating between the cervical and upper thoracic spine, these predispositions may act in concert with the unique anatomy of the cervicothoracic spine by further increasing the risk for wound dehiscence. Surgical wounds over the cervicothoracic spine are more susceptible to mechanical stress such as pressure and tension around the wound boarders, but also on the fascia due to high tensile and traction forces of the trapezius muscle [15]. Also, postoperative paraspinal muscle atrophy around the cervicothoracic junction will often lead to relatively more prominent spinous processes and implants compromising already poor skin conditions, especially in elderly patients. In this regard, a figure of eight bandage around the shoulders is an easy-to-apply measure, to reduce mechanical tear on the wound and to alleviate postoperative pain [16].

In order to reduce complication, SSI, and reoperation rates, a prophylactic coverage with muscle or fasciocutaneous flaps in spine patients at risk for postoperative wound healing disturbances has been advocated [14]. Chun and colleagues included a trapezius myocutaneous flap in the initial surgery to obliterate dead space and to cover spinal instrumentation in patients who had undergone previous radiation therapy [4]. A myocutaneous flap is especially well suited in defects with poorly vascularized tissue to provide a vital, well-perfused coverage.

The use of the transverse part of the trapezius muscle as a pedicled flap was first described by McCraw et al. [17] and Demegrasso and Piazza [18] in 1979, while the first description of the lower trapezius flap, using the ascending part of the muscle and its robust blood supply, is credited to Baeck et al. [19] and Mathes et al. [20]. Since then, the LTIMF has become a workhorse flap for reconstruction of soft tissue defects of the cervicothoracic spine (Fig. 1a). The technique was subsequently adapted using the lower part of the muscle for reconstruction of facial skin defects or for subcutaneous augmentation of the face [19]. The LTIMF proved to be a versatile surgical technique for various indications, mostly revolving around oromaxillofacial carcinoma therapy [21–29]. Eventually, the LTIMF and its variants were adapted to reconstructive spinal surgery. A case series in 1988 described this technique to cover complex wound dehiscence at the occipitocervical junction [10]. The LTIMF has since been shown to provide a stable and reliable coverage, even as a salvage therapy after failure of a prior pedicled flap [11].

### Limitations

The value of this report may be limited due to the small number of patients included in this case series. Still, larger series of spine patients managed with a LTIMF for complex wound healing disorders are lacking in the spine literature. Second, the clinical follow-up of our patients is rather short, and we therefore cannot evaluate the long-term results of LTIMF management in this study. Nonetheless, most likely, any flap failure in these patients beyond the last follow-up would have been brought to our attention.

## Conclusions

Salvage treatment using a LTIMF is a reliable and effective treatment option for patients with complex wound healing disorders at the cervical and thoracic spine. Under targeted antibiotic treatment—for culture-positive SSIs—and following VAC dressings, complicated myocutaneous defects can be successfully covered with a LTIMF, without the need of hardware removal. An interdisciplinary approach involving spine surgeons, plastic surgeons, and infectious disease specialists plays a critical role for successful management.

## Data Availability

All relevant clinical data is stored in our hospital information system, Inselspital, Bern, Switzerland.

## Declarations

Permission to reproduce copyrighted materials or signed patient consent forms is given. The manuscript submitted does not contain information about medical device(s)/drug(s).
